# Differential DNA methylation analysis across the promoter regions using methylated DNA immunoprecipitation sequencing profiling of porcine loin muscle

**DOI:** 10.14202/vetworld.2020.1113-1125

**Published:** 2020-06-16

**Authors:** Kaj Chokeshaiusaha, Denis Puthier, Thanida Sananmuang, Em-on Olanratmanee, Catherine Nguyen, Roongtham Kedkovid

**Affiliations:** 1 Department of Veterinary Science, Faculty of Veterinary Medicine, Rajamangala University of Technology Tawan-OK, Chon Buri, Thailand; 2 Aix-Marseille University, INSERM UMR 1090, TAGC, Marseille, France; 3 Department of Veterinary Medicine, Faculty of Veterinary Science, Chulalongkorn University, Bangkok, Thailand; 4 Swine Reproduction Research Unit, Chulalongkorn University, Bangkok, Thailand

**Keywords:** differential DNA methylation, lean, loin muscle, marbled, methylated DNA immunoprecipitation sequencing, transcription start site

## Abstract

**Background and Aim:**

Pork leanness and marbling are among the essential traits of consumer preference. To acquire knowledge about universal epigenetic regulations for improving breed selection, a meta-analysis of methylated DNA immunoprecipitation sequencing (MeDIP-seq) profiling data of mixed loin muscle types was performed in this study.

**Materials and Methods:**

MeDIP-seq profiling datasets of longissimus dorsi muscle and psoas major muscles from male and female pigs of Landrace and Tibetan breeds were preprocessed and aligned to the porcine genome. Analysis of differential methylated DNA regions (DMRs) between the breeds was performed by focusing on transcription start sites (TSSs) of known genes (−20,000-3000 bases from TSS). All associated genes were further reviewed for their functions and predicted for transcription factors (TF) possibly associated with their TSSs.

**Results:**

When the methylation levels of DMRs in TSS regions of Landrace breed were compared to those of Tibetan breed, 10 DMRs were hypomethylated (Landrace < Tibetan), and 19 DMRs were hypermethylated (Landrace > Tibetan), accordingly (p≤0.001). According to the reviews about gene functions, all associated genes were pieces of evidence for their roles in a variety of muscle and lipid metabolisms. Prediction of the binding TFs revealed the six most abundant binding TFs to such DMRs-associated TSS (p≤0.0001) as follows: ZNF384, Foxd3, IRF1, KLF9, EWSR1-FLI1, HES5, and TFAP2A.

**Conclusion:**

Common DMRs-associated TSS between the lean-type and the marbled-type loin muscles were identified in this study. Interestingly, the genes associated with such regions were strongly evidenced for their possible roles on the muscle trait characteristics by which further novel research topics could be focused on them in the future.

## Introduction

Pork has been accounted for a large-scale world’s meat consumption. Lately, the evolved preference of pork consumers significantly affects its amendment in taste, flavor, juiciness, and tenderness [1,2]. These issues thus become one of the most critical concerns in fields of both veterinary and animal health science as pork leanness and marbling are two essential traits contributing to its quality [1-3]. Diverse cascading gene transcription control by epigenetic modification was well-recognized for its involvement in such phenotypic variations among pig breeds [4-7]. The knowledge in epigenome concerning porcine musculogenesis and lipogenesis has thus significantly contributed to advance in swine breeding for decades [3-6,8]. For further explanation, epigenetic gene regulation by DNA methylation involves primarily *cis*-acting DNA elements which are the non-coding DNA binding sites for the gene transcription factors (TFs). Apart from the promoter region, which commonly locates near the transcription start site (TSS), the chromatin loop formation also allows the long-distant *cis*-acting elements such as enhancers and repressors to cooperate in regulation [9]. *Cis*-acting elements in mammals, including pigs, usually contain repeated CpG bases, by which their methylation can interfere TF binding. The methylation patterns are inheritable among animal breeds, including swine, and responsible for different transcription patterns among the tissues of interest [4-6,9,10]. To study the intricate regulation patterns of genome-wide DNA methylome, large-scale identification of enriched methylated sequences is thus required. Isolation of these methylated DNA fragments could be archived by the antibody against 5-methyl-cytosine, the purification technique recognized as methylated DNA immunoprecipitation (MeDIP) [11]. Coupling of MeDIP and short-read sequencing technologies even allow the high-throughput sequencing of methylated DNA fragments, the technology recognized as MeDIP-sequencing (MeDIP-seq) [12]. Despite its recent introduction, MeDIP-seq has been applied with a few cell types to discover differential DNA methylation patterns among different swine breeds [4,5,7,11,13]. Despite the insight into the epigenetic background of swine breeds implied by the past studies, some limitations in such knowledge’s application should also be concerned about.

DNA methylation profiling data of a tissue sample are a composited methylation pattern acquired from common cell types in the particular tissue [14]. Methylation profiling of each tissue type should not be considered as a good representative among one another [14,15]. Fortunately, differential DNA methylation analysis between lean-type and obese-type pig breeds was previously performed using the MeDIP-seq technology. With few replicated muscle tissue samples, a pooled library of one muscle type was constructed entirely for both DNA-seq and mRNA-seq analyses [4]. In the analysis of sequencing data, numbers of discrete biological replicate crucially influence representativeness of the differential analysis outcome [16,17]. By mean of this, the inclusion of numbers of different muscle type biosamples in differential methylation analysis should result in different significant patterns which are universal among various muscle-type tissues.

It should be noted that distances of *cis*-acting DNA elements from their corresponding TSSs could vary from few to further than 20 Kb [9,18]. While methylated elements are commonly presumed to regulate the transcription of the nearest gene promoter, their distant functions were also evidenced [19,20]. With clusters of different TFs possibly associated with these elements, they can complexly control the transcription of the targeted genes [19,20]. Virtualization of the methylated elements’ distances from the TSS and their associated TFs’ prediction is thus crucial for productive genome-wide DNA methylation profiling observation. Despite such feasibility, procedures to attain such knowledge in porcine muscle MeDIP-seq study were still limitedly demonstrated.

Gradual increase of porcine meat muscle MeDIP-seq profiling data in the public database has offered us an opportunity to demonstrate the analytical process with them. With available datasets acquired from two types of joint meat muscles, differential DNA methylation profiling analysis between lean and marbled meat pig breeds was performed in this study. Distance determination and TF prediction respectable to the regions were also demonstrated. By including numbers of different muscle tissue datasets, methylated DNA regions associated with the several well-recognized genes between lean and marbled meat muscle were successfully identified.

## Materials and Methods

### Ethical approval

All datasets used in this study were available in NCBI SRA public site, and no ethical approval was required.

### Sample datasets

The list of porcine meat muscle tissue MeDIP-seq profiling datasets is provided in Table-1. Longissimus dorsi muscle and psoas major muscles from both male and female pigs were included in this study. These muscle tissues were generated from two swine breeds: Landrace as the lean-type breed and Tibetan as the marbled-type meat breed.

**Table-1:** Porcine muscle tissue datasets used in this study.

Dataset	Breed	Sex	Muscle tissue
SRR307959	Landrace	Male	Longissimus dorsi muscle
SRR307960	Landrace	Male	Longissimus dorsi muscle
SRR307961	Landrace	Male	Longissimus dorsi muscle
SRR307962	Landrace	Male	Psoas major muscle
SRR307963	Landrace	Male	Psoas major muscle
SRR307964	Landrace	Male	Psoas major muscle
SRR307989	Landrace	Female	Longissimus dorsi muscle
SRR307990	Landrace	Female	Longissimus dorsi muscle
SRR307991	Landrace	Female	Longissimus dorsi muscle
SRR307992	Landrace	Female	Psoas major muscle
SRR307993	Landrace	Female	Psoas major muscle
SRR307994	Landrace	Female	Psoas major muscle
SRR308019	Tibetan	Male	Longissimus dorsi muscle
SRR308020	Tibetan	Male	Longissimus dorsi muscle
SRR308021	Tibetan	Male	Longissimus dorsi muscle
SRR308022	Tibetan	Male	Psoas major muscle
SRR308023	Tibetan	Male	Psoas major muscle
SRR308024	Tibetan	Male	Psoas major muscle
SRR308049	Tibetan	Female	Longissimus dorsi muscle
SRR308050	Tibetan	Female	Longissimus dorsi muscle
SRR308051	Tibetan	Female	Longissimus dorsi muscle
SRR308052	Tibetan	Female	Psoas major muscle
SRR308053	Tibetan	Female	Psoas major muscle
SRR308054	Tibetan	Female	Psoas major muscle

### Study period and location

The research was conducted at Department of Veterinary Science, Faculty of Veterinary Medicine, Rajamangala University of Technology Tawan-OK, Chon Buri, Thailand, from August 2019 until the end of January 2020.

### Analytical workflow

**Figure-1:**
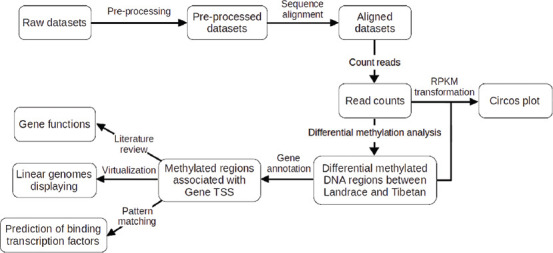
Analytical workflow for this study.

The analytical workflow of this study is shown in Figure-1. The preprocessed sample datasets were aligned to the porcine genome to acquire aligned datasets and counted for the methylated DNA sequence reads (read counts). Differential DNA methylation analysis between muscle datasets of Landrace and Tibetan pigs was performed. Both differential methylation results and read counts in Reads Per Kilobase Millions were visualized by the Circos plot (Figure-1). Differentially methylated DNA regions (DMRs) between Landrace and Tibetan pigs were subsequently determined for their proximity to TSSs of the associated genes (−20,000-3000 bases from TSS) and displayed by a customized plot (linear genomes displaying in Figure-1). All the associated genes were reviewed for their functions. Finally, TFs associated with these regions would then be predicted (Figure-1).

### Data preprocessing and quality assessment

The sequence read archive (SRA) files of porcine muscle MeDIP-Seq datasets were obtained from the SRA database (https://www.ncbi.nlm.nih.gov/sra) (Table-1). Data preprocessing was performed as previously described with some modifications [21]. Briefly, each dataset was aligned to the porcine reference genome (*Sus scrofa* UCSC susScr3) with the Bowtie2 aligner (version 2.3.4.3) [22]. Duplicated sequences of the aligned datasets were then removed [23]. Phred quality score was determined by FastQC [24]. The assessment of MeDIP-Seq quality and DNA methylation score was performed by Bioconductor package MEDIPS (250-bp windows) after extending read to a length of 300 bp [25].

### DMRs between landrace and Tibetan pigs

**Figure-2:**
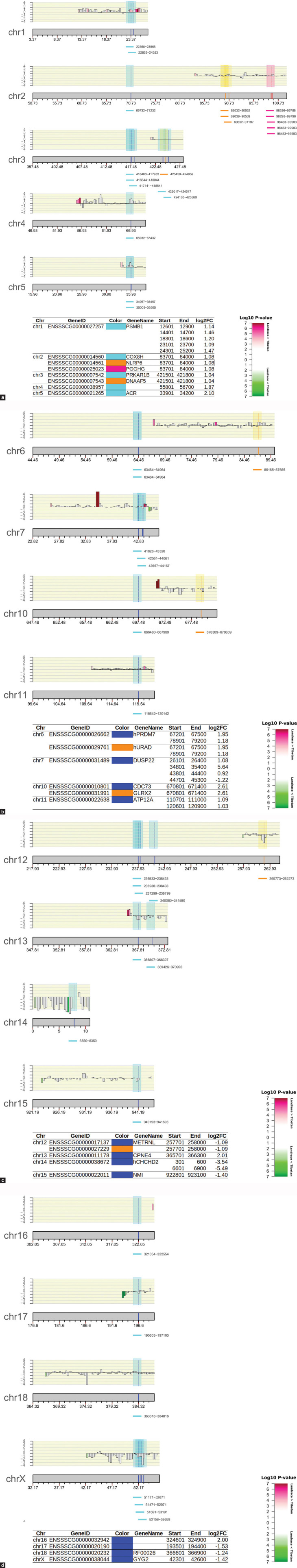
Linear genomes displaying methylated regions associated with gene transcription start sites (TSS). The karyoplot of 18 porcine genes with the presence of methylated DNA regions associated with their TSS (−20,000-3000) and promoter regions (−1000-500). Partial porcine genome sequences acquired from 17 chromosomes (chr) containing all associated gene transcripts were drawn with loci index (a-d). Promoter regions of transcripts presented on the same chromosome were demonstrated in different shaded colors – sky blue, orange, and pink, accordingly. Differentially hypomethylated to hypermethylated regions were demonstrated by gradient colors from deep blue to deep red respective to their −log_10_ p-values. The levels of differences were indexed by two-fold changes (− for hypomethylated and + for hypermethylated). The bottom legends indicated the Ensembl ID and common names of the targeted genes in each of the chr.

DMRs between Landrace and Tibetan pigs were identified (p≤0.001) by edgeR (exact test for negative binomial distribution) integrated with the MEDIPS package. TSSs of porcine genes were annotated using the porcine genome (Sscrofa 11.1) acquired from the Ensembl database (version 95) (https://asia.ensembl.org/info/data/ftp/index.html). This study only considered the DMRs within −20,000-+3000 bases from TSS. Gene promoter region was considered from −1000 to +500 bases from TSS and was exclusively remarked for the presence of differential methylation within the regions in the karyoplot (Figure-2).

### Prediction of binding TFs

RSAT Matrix-Scan (http://rsat.ulb.ac.be/) was used to detect TF binding sites (http://rsat.sb-roscoff.fr/) among the DMRs. Individual sites were predicted using the various position matrix of non-redundant TF binding profiles of vertebrates (JASPAR CORE, non-redundant vertebrates database) and filtered using a threshold on p=10^−6^. For matrix-scan, the first-order Markov chain background model was calculated from the input sequence set.

### Virtualization

Count reads of overall methylated DNA regions along with their DMRs between Landrace and Tibetan pigs were illustrated by the Circos plot using the Bioconductor OmicCircos package [26]. To display the DMRs associated with the target genes of interest, the karyoploteR package [27].was utilized to draw the plot for displaying the locations of DMRs and their associated TSSs.

## Results

### Genome-wide DNA methylation profiling and their differentially methylated DNA regions

**Figure-3:**
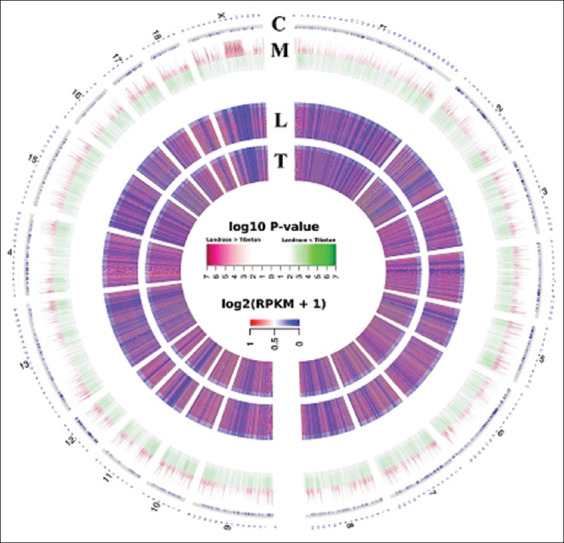
Circos plot of overall methylated DNA regions in the porcine genome. The circos plot demonstrated differentially methylated DNA regions among porcine chromosomes (C panel) (chr1-18 and chrX). Differences in methylation levels between Landrace and Tibetan pigs along each chromosome’s regions were manifested (M panel). The legend presented −log_10_ p-values of regions with gradient colors from red (higher methylation levels in Landrace) to green (higher methylation levels in Tibetan), accordingly. Log_2_(RPKM+1) values of count methylated regions acquired from Landrace (L panel) and Tibetan (T panel) pig breeds were shown. RPKM=Reads per kilobase millions.

After removing the contaminated, low quality, and adapter sequences, each preprocessed dataset had approximately 64 million clean reads (40-42 bps) with at least an 85% alignment rate to the *S. scrofa* reference genome. The data showed sufficient sequence depth, and coverage analyses revealed that approximately 95% of all CpGs in the *S. scrofa* genome was covered at least one-fold, whereas nearly 50% of CpGs were covered more than five-fold. Patterns of canonical epigenetic DNA methylation observed among longissimus dorsi muscle and psoas major muscle tissues acquired from both Landrace and Tibetan breeds were similar, by which strong positive correlation across the breed was demonstrated by correlation analysis (r=0.75-0.95, p<10^−16^). The comparison of the DNA methylation profiles of the Landrace and Tibetan breeds revealed 74,009 DMRs (p≤0.001) after merging significant neighboring windows. When compared, methylation levels of Landrace breed in these DMRs to those of Tibetan breed, 28,028 hypomethylated regions (Landrace < Tibetan) and 45,981 hypermethylated regions (Landrace > Tibetan) were found scattering among somatic and X chromosomes (Figure-3).

### Differentially methylated regions associated with TSS of porcine genes

The total of 29 DMRs, which were ten hypomethylated and 19 hypermethylated DMRs, was found distributed among the TSS regions (−20,000-+3000 from TSS) of 18 known genes (p≤0.001) (Figure-2a-d). Among these TSS-associated DMRs, seven DMRs consisting of one hypomethylated and six hypermethylated DMRs were presented in the gene promoter areas (−1000-+500 from TSS). The hypomethylated promoter region was contributed to the *ESSSCG00000038672* gene, which is orthologous to the human CHCHD2 gene (*hCHCHD2*) (chr15 in Figure-2c), while hypermethylated promoter regions were presented in *ESSSCG00000027257* (PSMB1) (chr1 in Figure-2a) and *ESSSCG00000031489* (DUSP22) (chr7 in Figure-2b). Two miRNA genes, *ENSSSCG00000020190* and *ENSSSCG00000020232* (RF00026) were identified (Figure-2d). Interestingly, subsequent gene function review manifested considerable affiliation of several protein-coding genes, except *ENSSSCG00000021265* (ACR), with the metabolism and function of muscle tissue (Table-2) [28-59]. The six most abundant TFs predicted to bind the DMRs in these TSS regions (p≤0.0001) were as follows: ZNF384 (56 times), Foxd3 (25 times), IRF1 (24 times), KLF9 (17 times), EWSR1-FLI1 (12 times), HES5 (12 times), and TFAP2A (12 times). For each DMR, the predicted binding TF with the highest significant score is displayed in Table-3.

**Table-2:** Methylated TSS associated genes with evidenced roles in muscle tissues.

Chr	GeneID	Common name	Function	References
chr1	ENSSSCG00000027257	PSMB1	Upregulated according to obesity, PSMB1 was transcriptional activator of RBP4 – a gene associated with insulin resistance and transcription activation of adipocyte.	[28,29]
chr2	ENSSSCG00000014560	COX8H	COX8H was related to cytochrome C oxidase – the crucial enzyme with potential role in oxidative and fatty acid metabolisms of muscle fibers.	[30,31]
	ENSSSCG00000014561	NLRP6	NLRP6 was the sensor component of NLPP3 inflammasome which could enhance muscular dystrophy and was strongly associated with obesity.	[7,32-34]
	ENSSSCG00000025023	PGGHG	PGGHG was a catalyzing enzymes of D-glucose, and thus could affect both muscle and adipose cell’s metabolisms.	[35,36]
chr3	ENSSSCG00000007542	PRKAR1B	PRKAR1B encoded a regulatory subunit of cyclic AMP-dependent protein kinase A in the signaling pathway of the second messenger cAMP – contributing to myofiber size and negatively associating with intramuscular fat.	[32,37]
chr6	ENSSSCG00000026662	hPRDM7	PRDM7 could function as a histone methyltransferase, and thus was likely affect DNA methylation affecting muscle tissue development and function.	[38]
	ENSSSCG00000029761	hURAD	URAD enzyme catalyzed anti-oxidant process of uric acid to form allantoin – the marker of oxidative stress in muscle.	[39,40]
chr7	ENSSSCG00000031489	DUSP22	DUSP22 protein activate the JNK signaling pathway – regulating the phosphorylation state of several kinases in skeletal muscle and requiring for obesity.	[41-44]
			DUSP22 was an obesity candidate gene which was hypermethylated in obese subject.	[45-47]
chr10	ENSSSCG00000010801	CDC73	CDC73 protein was a subunit of PAF protein complex associated with the RNA polymerase II subunit and a histone methyltransferase complex, and thus played role in chromatin modifications and gene transcription of muscle tissue.	[48]
	ENSSSCG00000031991	GLRX2	GLRX2 protein implicated MITOCHONDRIAL REDOX REGULATION preventing oxidative damage and was evidenced to control oxidative phosphorylation in cardiac muscles.	[49,50]
chr11	ENSSSCG00000022638	ATP12A	ATP12A was responsible for potassium absorption for muscle contraction – by which the lack of potassium could result in muscle destruction by decreased blood flow.	[51,52]
chr12	ENSSSCG00000017137	METRNL	METRNL product was associated with adipocyte browning and glucose tolerance.	[53]
	ENSSSCG00000027229		Several miRNAs in skeletal muscle could regulate myogenesis, hypertrophy, atrophy, and regeneration.	[54]
chr13	ENSSSCG00000011178	CPNE4	CPNE4 product was calcium-dependent phospholipid-binding protein, and thus can involve in several calcium-mediated processes in muscle tissue.	[55]
chr14	ENSSSCG00000038672	hCHCHD2	CHCHD2 was a transcription factor of cytochrome C oxidase required for optimal mitochondrial function of muscle cell.	[56]
chr15	ENSSSCG00000022011	NMI	N-myc can functionally replace c-myc in murine development, cellular growth, and differentiation.	[57]
chr16	ENSSSCG00000032942	DAP	DAP was necessary for complete myotube development during muscle fiber formation.	[58]
chrX	ENSSSCG00000038044	GYG2	Glycogenin was important for muscle glycogenesis – by which glycogen over-accumulation could result in fatigue reduced function of muscle.	[59]

**Table-3:** Top predicted transcription factors binding to differentially methylated TSS regions.

TSS regions	Transcription factor	Weight	p-value	*Significant score
chr1:12601-12900	EGR4	8.7	1.6E-05	4.796
chr1:14401-14700	ZSCAN4	8.3	6.3E-06	5.201
chr1:18301-18600	GATA1::TAL1	10.1	8.1E-06	5.092
chr1:23101-23700	TEAD1	9.3	2.3E-06	5.638
chr1:24301-25200	ZNF143	9.1	3.2E-06	5.495
chr10:670801-671400	Foxd3	10.3	3.5E-06	5.456
chr11:110701-111000	KLF9	11.6	5.8E-07	6.237
chr11:120601-120900	Dmbx1	10.7	3.2E-06	5.495
chr12:257701-258000	ZNF384	10.2	4.8E-07	6.319
chr13:365701-366300	ZNF384	10.1	1.1E-06	5.959
chr14:10801-11100	NFYA	9.6	1.2E-05	4.921
chr14:26401-26700	SPI1	4.7	2.9E-05	4.538
chr14:301-600	CTCF	14.2	6.7E-08	7.174
chr14:6601-6900	Foxd3	11	9.1E-07	6.041
chr15:922801-923100	TBP	10.7	1.5E-06	5.824
chr16:324601-324900	RBPJ	9.2	1E-05	5
chr17:193501-194400	NFIL3	10	8.4E-06	5.076
chr18:366601-366900	EWSR1-FLI1	6.8	1.3E-06	5.886
chr2:83701-84000	RELB	8.9	2E-05	4.699
chr3:421501-421800	SNAI2	8.7	1.7E-06	5.77
chr4:55801-56700	HSF4	11.4	1.4E-06	5.854
chr5:33901-34200	HIC2	8.4	1.1E-05	4.959
chr6:67201-67500	Dlx2	8.3	7.5E-06	5.125
chr6:78901-79200	Arid5a	10.1	3.1E-06	5.509
chr7:26101-26400	SP2	12.4	4.2E-07	6.377
chr7:34801-35400	Stat5a::Stat5b	9.9	5.7E-06	5.244
chr7:43801-44400	HIF1A	11.8	1.6E-07	6.796
chr7:44701-45300	KLF5	11.2	8.7E-07	6.06
chrX:42301-42600	Klf1	11.1	1.7E-06	5.77

* Significant score = −log10 (p-value x nb.words). TSS=Transcription start sites

## Discussion

DNA methylation patterns mutually distributed among the tissue types usually implied universal ­metabolic features shared in common among them [14]. Based on such an approach, this study included considerable MeDIPS datasets generated from two common loin meat muscle tissues, longissimus dorsi muscle and psoas major, with the differential methylation analysis to discover the universal differences in DNA methylation pattern of loin muscle to understand the differences between lean and marbled meat pig breeds. Since only rigid cellular metabolisms were controlled in universal patterns among tissue types, limited numbers of genes with differentially methylated TSS regions were expected as successfully demonstrated in this study.

Several novel genes with TSS-associated DMRs were also exclusively identified in this study. Apart from constructing sample libraries from different meat tissues, other factors, such as muscle types, dataset numbers, and pig breeds, were also considered as contributors to such achievement (Figure-2). For the available virtualization of these regions’ localization following their associated genes, the karyoplot was successfully applied [27]. Among all the presented TSS-associated DMRs, some DMRs in the promoter regions of three coding genes, *ESSSCG00000038672* (*hCHCHD2*), *ESSSCG00000027257* (*PSMB1*), and *ESSSCG00000031489* (*DUSP22*) were also noticeable (Figure-2a-c). Due to the robust influence of promoter on epigenetic gene regulation [9,19]. these differentially methylated genes should be considered as exclusive candidates for further transcription determination. Of note, the current study only focused on TSS-associated methylated regions. Further analysis of other potential epigenetic gene regulation regions, including gene exons [60] and introns [61,62], was thus encouraged in separated studies.

In this study, several novel genes in loin muscles manifested their differences in the TSS region methylation levels in Landrace comparing to Tibetan pigs. Landrace pig is recognized for its lean large muscle bundle, while Tibetan pig muscle is more compact with outstanding marbling levels [63]. The different characteristics of the two breeds thus greatly denoted the diverge metabolisms of their muscle tissues. Supporting this, almost all differentially methylated genes also manifested their evidenced functions relating to musculogenesis and lipid metabolisms in this study (Table-2). Interestingly, two differentially methylated miRNA genes, *ENSSSCG00000020190* and *ENSSSCG00000020232*, were also identified (Figure-2d), contributing new targets for a miRNA study in the skeletal muscles [54].

The regulatory function of the acquired TSS-associated DMRs required further biological evaluation despite the knowledge of their methylation levels. Since DNA methylation could either inhibit or recruit particular TFs in various circumstances [9,20,64]. the prediction of candidate TFs binding regions was thus crucial to study their epigenetic gene regulation. Due to the limited demonstration of such process in porcine skeletal muscle MeDIP-seq study, we introduced the RSAT Matrix-Scan as a convenient web-based tool for the prediction of TF binding. Of note, this study intentionally showed only TFs with the highest prediction significant score for demonstration. Since each TSS region could be occupied by various TFs [65]. other significantly predicted TFs (p≤10^−6^) should also be considered for any further intensive study.

## Conclusion

In summary, the determination of TSS regions that were differentially methylated between lean-type and marbled-type pig breeds in loin meat muscle tissues was demonstrated as a novel approach in this study. The procedure rendered the limited numbers of differentially methylated genes, but with strong concatenation with the different muscle characteristics presented between these two breeds. We also introduced a karyoplot for the available virtualization of TSS-associated DMRs along with their candidate TFs binding to them. As a result, the knowledge acquired from this study established several new research topics in lean and marbled meats to evaluate the epigenetic gene regulation. It is worth noting that this study aimed to demonstrate a beneficial strategy for porcine muscle MeDIP-Seq data analysis, which was also applicable to other biological systems.

## Authors’ Contributions

KC, TS and RK planned the study design, collected the datasets, and analyzed the data. EO and CN refined the study design and the objective. DP carried out technical coding correction and hardware maintenance. KC and RK drafted and reviewed the manuscript. All authors read and approved the final manuscript.
